# Prophylaxis with abemaciclib delays tumorigenesis in dMMR mice by altering immune responses and reducing immunosuppressive extracellular vesicle secretion

**DOI:** 10.1016/j.tranon.2024.102053

**Published:** 2024-07-09

**Authors:** Annabell Wolff, Paula Krone, Johanna Maennicke, Julia Henne, Sonja Oehmcke-Hecht, Caterina Redwanz, Wendy Bergmann-Ewert, Christian Junghanss, Larissa Henze, Claudia Maletzki

**Affiliations:** a Department of Medicine, Clinic III –Hematology, Oncology, Palliative Medicine, Rostock University Medical Center, University of Rostock, 18057 Rostock, Germany; b Institute of Medical Microbiology, Virology and Hygiene, Rostock University Medical Center, University of Rostock, 18057 Rostock, Germany; c Department of Internal Medicine B, Cardiology, University Medicine Greifswald, Germany; d Core Facility for Cell Sorting & Cell Analysis, Laboratory for Clinical Immunology, Rostock University Medical Centre, 18057, Rostock, Germany

**Keywords:** Hereditary cancer prevention, Cell cycle regulation, Immune modulation, Clotting

## Abstract

**Background:**

The CDK4/6 inhibitor abemaciclib is an FDA-approved agent and induces T-cell-mediated immunity. Previously, we confirmed the therapeutic potential of abemaciclib on mismatch repair-deficient (dMMR) tumors in mice. Here, we applied a prophylactic administration/dosage setting using two preclinical mouse models of dMMR-driven cancer.

**Methods:**

Mlh1^−/−^ and Msh2^loxP/loxP^ mice received repeated prophylactic applications of abemaciclib mesylate (75 mg/kg bw, per oral) as monotherapy or were left untreated. Blood phenotyping and multiplex cytokine measurements were performed regularly. The tumor microenvironment was evaluated by immunofluorescence and Nanostring-based gene expression profiling. Numbers, size and immune composition and activity of extracellular vesicles (EVs) were studied at the endpoint.

**Findings:**

Prophylactic abemaciclib-administration delayed tumor development and significantly prolonged overall survival in both mouse strains (Mlh1^−/−^: 50.0 wks vs. control: 33.9 wks; Msh2^loxP/loxP;TgTg(Vil1-cre^: 58.4 wks vs. control 44.4 wks). In Mlh1^−/−^ mice, pro-inflammatory cytokines (IL-2, IL-6) significantly increased, whereas IL-10 and IL-17A decreased. Circulating and splenic exhausted and regulatory T cell numbers were significantly lower in the abemaciclib groups. Deeper analysis of late-onset tumors revealed activation of the Hedgehog and Notch signaling in Mlh1^−/−^ mice, and activation of the MAPK pathway in Msh2^loxP/loxP;TgTg(Vil1-cre^ mice. Still, arising tumors had fewer infiltrating myeloid-derived suppressor cells (vs. control). Notably, prophylactic abemaciclib-administration prevented secretion of procoagulant EVs but triggered release of immunomodulatory EVs in Mlh1^−/−^ mice.

**Interpretation:**

Prophylactic abemaciclib prolongs survival *via* global immunomodulation. Prophylactic use of abemaciclib should be considered further for individuals with inherited dMMR.

**Funding:**

This work was supported by grants from the German research foundation [DFG grant number: MA5799/2–2] and the Brigitte und Dr. Konstanze Wegener-Stiftung to CM.

## Highlights

• Preventive CDK4/6 inhibition with abemaciclib significantly delays tumor development in preclinical hereditary cancer models.
• abemaciclib alters immune responses and preserved an proinflammatory immune status.
• abemaciclib administration prevents secretion of procoagulant extracellular vesicles but triggers release of immunomodulatory extracellular vesicles.

## Introduction

The FDA-and EMA-approved selective cyclin-dependent kinase (CDK) 4/6 inhibitor abemaciclib is currently standard of care for HR-positive, HER2-negative metastatic breast cancer in the adjuvant and metastatic setting [1,2]. CDKs and cyclins are cell cycle regulators [3]. CDK4 and CDK6, together with cyclin D, phosphorylate the retinoblastoma suppressor protein. This leads to cell cycle progression from G1 to S phase. Abemaciclib targets CDK4/6, resulting in G1 arrest and inhibition of the cell cycle progression [[4], [5], [6], [7]]. In addition, abemaciclib may enhance the immunogenicity of tumor cells by increasing lymphocyte infiltration, suppressing regulatory T cells (Treg), and recruiting cytotoxic T cells to the tumor microenvironment [8].

Mismatch repair deficiency (dMMR) is caused by hypermethylation of the *MLH1* gene promoter (= sporadic form) or by germline MMR mutations (= inherited form), especially *MLH1* and *MSH2* [9,10]. Developing dMMR tumors are highly immunogenic due to an increased mutation load forming cytotoxic T cell neoantigens. This finding contributed to the approval of immune checkpoint inhibitors as efficacious therapeutics for dMMR cancers, that are meanwhile clinical standard of care [11,12]. In some cases, however, T cell responses against subclonal neoantigens are blunted, contributing to immune evasion and, ultimately, treatment failure [13]. Hence, the identification of reliable predictive biomarkers constitutes an unmet need. In addition to the well-studied immune checkpoint molecules (such as PD-L1, CTLA-4, LAG-3), dMMR tumors are capable of releasing extracellular vesicles (EVs). They can be classified into exosomes, microvesicles and apoptotic bodies and differ in their origin, properties, and content (proteins, lipids) [14]. Recent studies have described the immunomodulatory effects of EVs by upregulating PD-L1 on tumor-associated macrophages (TAMs), influencing the differentiation and polarization of tissue-resident monocytes/macrophages into M1- and M2-subtypes, suppressing the activity of dendritic cells, and promoting the differentiation of monocytes into myeloid suppressor cells [15,16]. Moreover, tumor-derived EVs induce Treg cells *via* immunoregulatory molecules (TGF-ß, TRAIL) and may have procoagulant effects [15,17] [19,20]. The latter is the result of tissue factor (TF = activator of the extrinsic pathway) and phosphatidylserine (PS, co-activator) on the surface of the tumor cells, which increases the risk of thromboembolic complications [[18], [19], [20]].

Together, these facts call for preventive approaches in patients with a known hereditary cancer risk. Based on our previous studies demonstrating the antitumor potential of abemaciclib in the therapeutic setting [21], we investigated its immunomodulatory and thus tumor preventive activity in two preclinical dMMR-driven mouse models. Besides, we investigated the immunomodulatory and procoagulant properties of EVs as potential circulating and thus non-invasive biomarkers.

We could show that prophylactic abemaciclib delayed tumor formation and significantly prolonged overall survival *via* immunomodulatory effects.

## Methods

### Ethical statement

The German local authority approved all animal experiments: Landesamt für Landwirtschaft, Lebensmittelsicherheit und Fischerei Mecklenburg‐Vorpommern (7221.3‐1‐062/19; −025/20), under the German animal protection law and the EU Guideline 2010/63/EU. Mice were bred in the animal facility of the University Medical Center in Rostock under specific pathogen‐free conditions. Mlh1 genotyping was done according to [22,23] and Msh2 genotyping was done according to [24,25]. During their whole life-time, all animals received enrichment in the form of mouse-igloos (ANT Tierhaltungsbedarf, Buxtehude, Germany), nesting material (shredded tissue paper, Verbandmittel GmbH, Frankenberg, Deutschland), paper roles (75 × 38 mm, H 0528–151, ssniff‐Spezialdiäten GmbH), and wooden sticks (40 × 16 × 10 mm, Abedd, Vienna, Austria). During the experiment, mice were kept in type III cages (Zoonlab GmbH, Castrop‐Rauxel, Germany) at 12‐h dark:light cycle, the temperature of 21 ± 2 °C, and relative humidity of 60 ± 20 % with food (pellets, 10 mm, ssniff‐Spezialdiäten GmbH, Soest, Germany) and tap water ad libitum. When mice were subjected to treatment (= age <12 weeks), they were given daily-prepared soaked pellets to ensure proper food intake.

### Experimental protocol

Mlh1^−/−^ or Msh2^loxP/loxP;TgTg(Vil1-cre)^ mice without clinical signs of tumor development and aged <12 weeks received prophylactic abemaciclib mesylate applications (75 mg/kg bw, per oral, *n* = 14 mice/strain, 8 times in total, every 42 days: q42dx8). Control mice received no treatment (*n* = 14 mice/strain). The general health status was monitored daily. Mice were sacrificed based on human endpoints (weight loss >15%, pain, changes in social behavior) or when the maximum follow-up time was reached (15 months after 1st treatment). Blood, spleen, tumors, and bone marrow were collected for subsequent analyses.

### Blood and spleen immune phenotyping

Blood samples were routinely collected from anaesthetized mice (retrobulbar venous plexus) and centrifuged. Plasma was frozen by −80 °C. Spleen and tumor tissues were dissociated as described before [23,26]. Single cells were stained with a panel of conjugated monoclonal antibodies (mAb, 0.125 μg to 1.5 μg each) as described before [26,27]. Flow cytometry measurements were performed on a spectral flow cytometer (3L-Cytek™ Aurora). Data were analyzed using SpectroFlo™ Version 2.2.0.3. and FlowJo™ Version 10.6.1.

### Characterization of bone marrow-derived hematopoietic stem cells

The bone marrow was obtained by flushing the bones. Then, antibody staining was done to characterize hematopoietic stem cells as described [27]. Flow cytometry measurements were performed on a spectral flow cytometer (3L-Cytek™ Aurora). SpectroFlo™ version 2.2.0.3 was used for analysis and FlowJo™ version 10.6.1 for data evaluation.

### Multiplex cytokine assay

Plasma cytokine levels were quantified using a multi-analyte flow assay kit following the manufacturer's instructions (LEGENDplex™, Biolegend). Measurements were performed on a spectral flow cytometer (3L-Cytek™ Aurora). Data were analyzed using the manufacturer's online Software. Absolute plasma cytokine levels are presented [ng/ml].

### Nanostring targeted gene expression profiling

Ten 20 µm cryostat sections were collected and RNA was isolated using the RNeasy Mini Kit (Qiagen). The RNA concentration was measured by Nanodrop and was adjusted to a specific concentration. The analysis of RNA was performed by Nanostring analysis as described before [28].

### Immunofluorescence

The tumor microenvironment was studied as described before [[27], [28], [29]]. Additionally, Ki-67 was included as proliferation marker. Therefore, slides were stained with Alexa Fluor® 647 anti-mouse/human Ki-67 Antibody (1:50, overnight, Biolegend), followed by DAPI staining for nuclear staining.

### EV isolation from mouse EDTA plasma

Plasma was thawed and diluted with PBS up to 1 ml. The plasma was centrifuged three times at 14,000 x g for 30 min. Between every centrifugation step, 900 µl of the supernatant was removed and same amount of PBS was added. EVs were resuspended in 100 µl and used for subsequent experiments.

### Nanoparticle tracking analysis (NTA)

EV quantity, size, and concentration were measured using NanoSight® Ltd. (Amesbury, GB). The EVs were diluted with PBS and placed in the assembled measurement chamber. In addition, a thermometer was connected to the measurement chamber. During the measurement, five videos of 60 s each were recorded. The mean value of the concentration as well as the size of the most abundant EV population was calculated from all videos. To exclude particles of PBS, this was also measured and then subtracted from the samples.

### Clotting time assay

The coagulometer was preheated to 37 °C. The clotting time of the EVs was measured after the addition of mouse plasma and calcium chloride (CaCl_2_).

### Mouse TF ELISA

For the detection of TF on the extracellular vesicles, plasma from abemaciclib treated and control mice was analyzed using a mouse TF ELISA Kit from the company Assay Genie. The assay was performed according to the manufacturer's instructions.

### Co-culture of EV and mouse splenocytes

Mouse splenocytes from heterozygous Mlh1 mice were adjusted to 1 × 10^6^ in 5 ml, seeded into 6-well plates and then incubated for 24 h at 37 °C in an incubator. The medium was removed and EVs were adjusted to a 100-fold concentration of the cells. They were added to the cells and incubated for 48 h. Finally, the previously described immunophenotyping was performed using the 3L-Cytek™ Aurora.

### Sorting and reactive oxygen species (ROS)-Production of MDSCs and total splenocytes

Total splenocytes and sorted CD11b^+^GR1^+^ cells (= MDSC) from control and abemaciclib-exposed mice were measured after overnight stimulation with lipopolysaccharide (LPS) as described [27]. Briefly, ROS-Brite reagent was added and incubated at 37 °C for 20 min. Fluorescence measurement was performed using the Tecan Reader 200 Infinite pro (Ex/Em: 651/670 nm).

### Statistics

GraphPad PRISM software, version 8.0.2 (GraphPad Software, San Diego, CA, USA) was used to perform statistical evaluation. The value of significance was set to *p* < 0.05. Data was first tested for normality conducting Shapiro-Wilk test. In case of normality, one-way ANOVA (Tukey´s multiple comparison) or unpaired *t*-test was accomplished. If normality failed, Kruskal-Wallis or *U test* was applied. Kaplan Meier survival curves were analyzed using the log rank (Mantel Cox) test. In case of blood phenotyping, outliers were eliminated, when they were above or below the average plus/minus two times the standard deviation.

## Role of the funding source

This work was supported by grants from the German research foundation [DFG grant number: MA5799/2–2] and the Brigitte und Dr. Konstanze Wegener-Stiftung to CM.

## Results

### Abemaciclib prolongs overall survival of dMMR mice

**Fig. 1:**
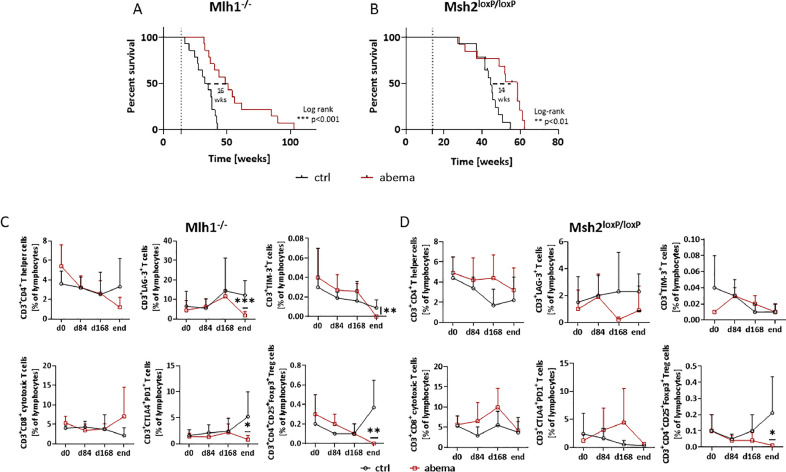
Overall survival and longitudinal spectral flow cytometry of the peripheral blood from Mlh1^−/−^ and Msh2^loxP/loxP^ mice that received abemaciclib (abema) in a prophylactic setting. (A, B) Kaplan Meier survival curve of both mouse strains. (A) *n* = 14 mice/group; (B) control *n* = 13, abema *n* = 11. ** *p* < 0.01; *** *p* < 0.001. Log-rank (Mantel Cox) analysis. (C, D) For spectral flow cytometry blood was taken routinely from retrobulbar venous plexus. A panel of surface and intracellular staining monoclonal antibodies was used and staining was done as described in material and methods. Mean + SD, (C) *n* = 4 – 9 mice/group; (D) *n* = 4 – 10 mice/group. (C, D) * *p* < 0.05; ** *p* < 0.01; *** *p* < 0.001 *vs*. control; Unpaired t-test.

In both mouse models, prophylactic application of abemaciclib significantly prolonged overall survival (Mlh1^−/−^: 50.0 *vs*. control: 33.9 wks; Msh2^loxP/loxP;TgTg(Vil1-cre^: 58.4 *vs*. control: 44.4 wks) (Fig. 1A, B). In Mlh1^−/−^ mice, the overall survival (OS) was extended by 16 weeks and in Msh2^loxP/loxP^ mice, OS was 14 weeks longer than in control mice. One Mlh1^−/-^ mice remained tumor-free until the end of follow-up (= 12 month after the last application). The remaining mice developed the typical tumor spectrum, i.a. lymphomas or gastrointestinal tumors (GIT). All Msh2^loxP/loxP^ mice developed GIT and were euthanized based on human endpoints. Autopsy confirmed progressive disease in virtually all cases (Table 1).

**Table 1:** Overview of clinicopathologic findings and eligibility for euthanasia.

Mouse number	Mlh1^−/−^		Msh2^loxP/loxP^	
	ctrl	abemaciclib	ctrl	abemaciclib
#1	Lymphomagenesis (generalized)	Lymphomagenesis (generalized)	Poor general condition/unknown cause	Weight loss/ PD/GIT
#2	PD/GIT	No tumor, ethical reasons (open wound at the back)	PD/GIT	Unexpected death
# 3	Lymphomagenesis (generalized)	GIT	PD/GIT	PD/GIT
# 4	GIT-induced intussusception	Analprolaps, no tumor	PD/GIT	PD/GIT
# 5	Lymphomagenesis (generalized)	GIT	PD/GIT	Several GIT
# 6	GIT	Suspected tumor growth (found dead)	PD/GIT	PD/GIT
# 7	GIT-induced intussusception	Ileus, GIT	PD/GIT	PD/GIT
# 8	GIT	GIT	PD/GIT	PD/GIT
# 9	GIT	GIT	PD/GIT	Ascites/GIT
#10	GIT	Lymphomagenesis (generalized)	PD/GIT	Weight loss
# 11	Lymphomagenesis (generalized)	GIT	PD/GIT	PD/GIT
# 12	Lymphomagenesis (generalized)	Lymphomagenesis (generalized)	PD/GIT	GIT
# 13	Lymphomagenesis (generalized)	Lymphomagenesis (lymph node)	PD/GIT	PD/GIT
# 14	Lymphomagenesis (generalized)	End of follow-up	PD/GIT	PD/GIT

PD – progressive disease, GIT – gastrointestinal tumor.

Longitudinal immune phenotyping partially confirmed the beneficial effect of abemaciclib on immune modulation in the blood (Fig. 1C, D). In Mlh1^−/−^ mice, T cell levels (CD3^+^CD4^+^ and CD3^+^CD8^+^) showed an inverse trend with increasing cytotoxic T cell numbers at the experimental endpoint. In contrast, T cell exhaustion remained low and Treg numbers gradually decreased (Fig. 1C). Supplementary cytokine level measurements confirmed these findings (supplementary Figure 1). Plasma cytokine levels dynamically changed and longitudinal measurements identified significantly higher amounts of IL-2 and IL-6 in abemaciclib-treated mice at day 84 (*p* < 0.05 vs. control), while IL-10 and IL-17A were significantly lower (*p* < 0.05 vs. control). TNFα and IL22 remained largely unchanged over the time course. In Msh2^loxP/loxP^ mice, peripheral immune phenotyping did not reveal such a uniform picture. The number of T cells was comparable to controls, and T cell exhaustion markers fluctuated over time (Fig. 1D). The only exception was the number of Treg cells, which showed a similar trend as in Mlh1^−/−^ mice and continuously decreased.

However, in both mouse strains we observed long-term immune-modulatory effects after repetitive abemaciclib applications that were evident at later time points and not directly visible during short-term prophylaxis.

### Abemaciclib reduces T cell exhaustion & ROS production in MLH1^−/−^, but not in Msh2^loxP/loxP^ mice

**Fig. 2:**
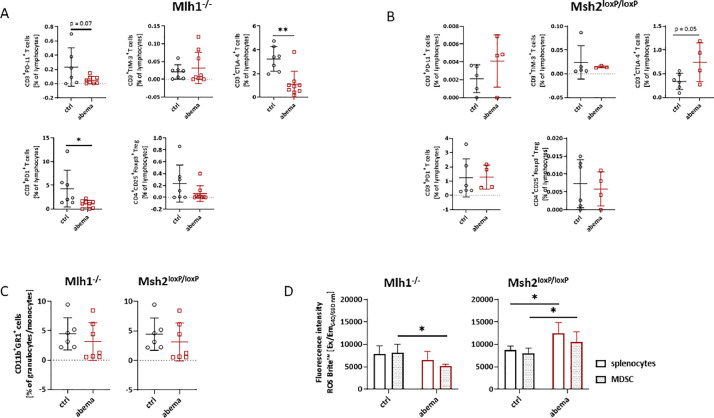
**Spectral flow cytometry of spleens.** (A, B, C) Given is the number of% immune cells at the experimental endpoint resulting from 100,000 events measured on a spectral flow cytometer. Mean + SD. (A) *n* = 5 – 9 mice/group; (B) *n* = 4 –6 mice/group. * *p* < 0.05; ** *p* < 0.01 *vs*. control; Mann Whitney test. (D) CD11b^+^Gr1^+^ sorted splenocytes were stimulated over night with lipopolysaccharide from *E. coli* (100 ng/ml). ROS production from unsorted and CD11b^+^Gr1^+^ sorted splenic MDSCs was determined using ROS Brite™ 670. Fluorescence intensity was measured on a preheated Tecan plate reader. Mean + SD, * *p* < 0.05 vs. control. Unpaired t-test.

Then, we focused on specific T cell exhaustion markers and Treg cells in the spleen. For the former, we focused on the immune checkpoint molecules PD-L1, TIM-3, CTLA-4, and PD-1 (Fig. 2A, B).

In both mouse models, a decrease in the number of regulatory T cells was observed. However, there were differences in markers of T cell exhaustion. In Mlh1^−/−^ mice, abemaciclib significantly reduced PD1^+^ and CTLA4^+^ *T* cells (*p* < 0.05 vs. control). Number of CD3^+^T cells expressing PD-L1 was also reduced (Fig. 2A). In contrast, abemaciclib-treated Msh2^loxP/loxP^ mice showed an increase in CD3^+^ *T* cells expressing the immune checkpoints PD-L1 and CTLA4. The remaining T cell exhaustion markers were nearly comparable to control mice (Fig. 2B). Hence, both mouse strains responded individually to abemaciclib in terms of T cell exhaustion.

Although MDSC numbers were largely unaffected by abemaciclib (Fig. 2C), we investigated whether the function of total splenocytes and CD11b^+^Gr1^+^-sorted MDSCs might differ. ROS production was analyzed after overnight culture and stimulation with LPS (Fig. 2D). Given the fact that sorting may have activated MDSCs in the spleen, a comparative approach was performed with total splenocytes and sorted MDSCs. In this experiment, opposite results were obtained in both mouse strains. In MLH1^−/-^ mice, we observed the expected reduced ROS production mainly in MDSCs (*p* < 0.05 vs. control). However, in Msh2^loxP/loxP^ mice, total splenocytes and purified MDSCs produced significantly more ROS than cells from untreated control mice. This is somewhat unexpected. However, it is consistent with the generally poorer response to abemaciclib in this mouse strain.

### Long-term effects of abemaciclib on the tumor microenvironment

**Fig. 3:**
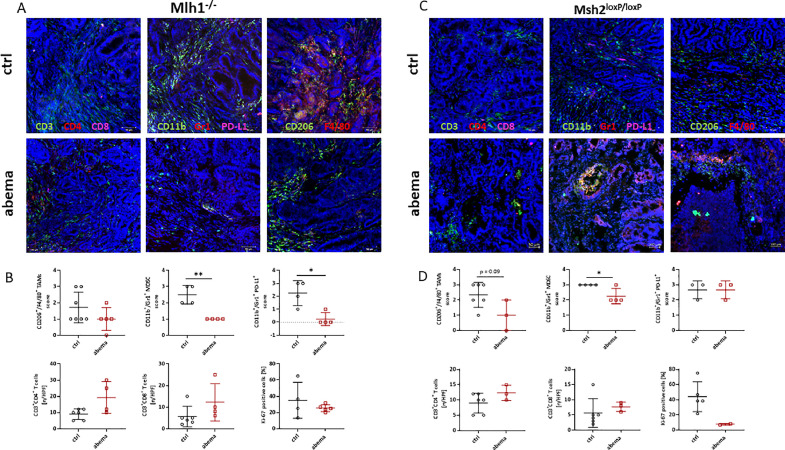
**Characterization of the TME using immunofluorescence.** The infiltration pattern of T cells and regulatory, and tumor-associated (M2) macrophages was examined in residual tumor slides. Confocal laser scanning microscopy was done on a Zeiss Elyra 7 microscope. (A, C) Representative images of tumor slides from both mouse strains. (B, D) Quantitative analysis of tumor-infiltrating immune cells counted in 2–3 HPFs/slide. Ki-67 positivity was scored in 2–3 HPFs/slides and the percentage of positive cells is given. Each symbol represents one mouse. *n* = 2 – 6 mice/group; Mean + SD, * *p* < 0.05; ** *p* < 0.01 *vs*. control; Mann Whitney test.

We then studied the impact of abemaciclib on the microenvironment of developing tumors (Fig. 3). In both mouse strains, the spatial distribution of TAMs, MDSCs, and regulatory granulocytes within tumors remained lower in treated mice than in controls. Consistently, tumors were more frequently infiltrated with T cells, i.e. CD3^+^CD4^+^helper and CD3^+^CD8^+^cytotoxic cells, while Tregs were unaffected (Fig. 3 and supplementary Figure 2A). Hence, prophylactic administration of abemaciclib has long-term immunomodulatory effects even after tumor progression. To study the proliferative capacity in more detail, we performed Ki-67 staining. Notably, in both mouse strains, the number of Ki-67 positive cells was lower in tumors that were pretreated with abemaciclib. Unfortunately, tumor tissue was not available in all cases and, therefore, statistical significance was not achieved. Still, we confirm here the long-term effects of the CDK4/6 inhibitor abemaciclib on dMMR-driven tumors in mice (Fig. 3).

**Fig. 4:**
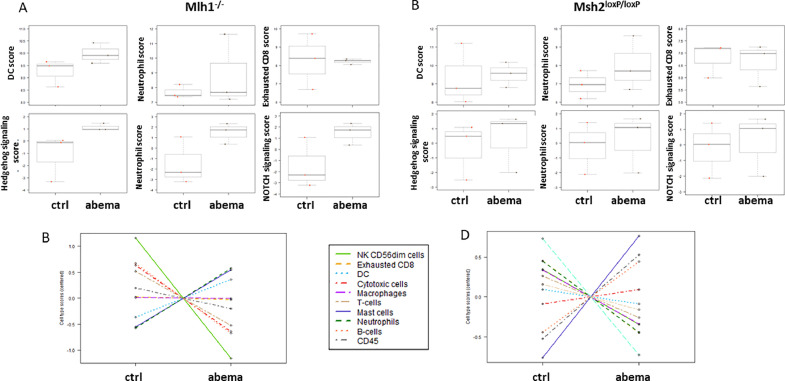
**Nanostring-based gene expression assay of tumors.** The PanCancer IO 360 Gene Expression Panel was applied. Relative abundances measuring various differences between cell types reported for each group. Data result from *n* = 3 samples/group. (A, B) Changes in specific cell types or signaling pathways according to gene expression data. (C, D) Analysis of specific cell types in control and abemaciclib-pretreated tumors.

Nanostring gene expression analysis (Fig. 4 and Supplementary Figure 2) provided further insight into the tumor microenvironment (TME). Interestingly, dendritic cells were more abundant in the abemaciclib groups, notably, in tumors of both MLH1^−/-^ and Msh2^loxP/loxP^ mice. Besides, neutrophils and mast cells were increased while T cell exhaustion levels were unaffected. Furthermore, the spatial distribution of different immune cell subsets differed between the two mouse models (Fig. 4C, D).

We then focused on deregulated pathways assuming them as the cause of final tumor development. MLH1^−/-^ tumors showed activation of the Hedgehog (i.e. 4.8-fold upregulation of *Gli1, p* = 0.05 *vs*. control) and Notch (i.e. 3.4-fold upregulation of *Jag2* and *Hey1, p* = 0.05 and 0.06 *vs*. control, respectively) signaling pathways (Fig. 4A), which was not seen in tumors from Msh2^loxP/loxP^ mice. Here, upregulation of genes involved in angiogenesis (i.e. 3.0-fold upregulation of *Fstl3* and *Fgfr1, p* = 0.08 and 0.1 *vs*. control, respectively) and activation of the MAPK pathway (i.e. 4.3-fold upregulation of *IL1r2, p* = 0.05 *vs*. control) were identified (supplementary Figure 2B). The Wnt pathway was not altered in any group (supplementary Figure 2B).

Taken together, these data suggest a compensatory activation of pro-malignant pathways that ultimately contributes to tumor progression following the prophylactic use of abemaciclib.

### Abemaciclib has minor impact on bone marrow hematopoiesis

**Fig. 5:**
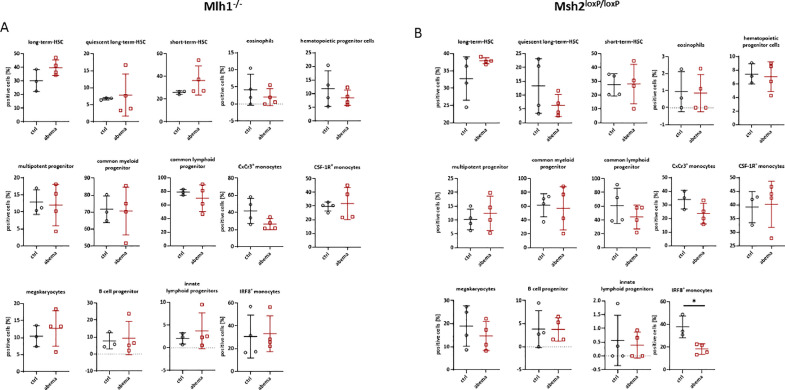
**Impact of abemaciclib on murine haematopoiesis.** A 20-marker panel was applied as described in material & methods. (A, B) Quantitative analysis of lin^−^*c*-Kit^+^ precursors according to a predefined characterization scheme [30]. Results show data from 100,000 cells/sample. *n* = 4 mice/group; Mean + SD, * *p* < 0.05; Mann Whitney test.

Tumor growth is associated with gross changes in hematopoiesis. In order to follow these changes after abemaciclib treatment, and to study whether prophylactic use of this agent may have affected hematopoiesis, we used an in-house hematopoietic stem cell full-spectrum flow cytometry panel (Fig. 5). In both mouse strains, abemaciclib increased the number of c-Kit^+^Sca-1^+^CD150^+^ long-term hematopoietic stem cells (long-term-HSC) after treatment. In contrast, the number of quiescent long-term-HSCs and CxCr3^+^ monocytes decreased. Apart from this, no gross changes were observed. An exception was seen in Msh2^loxP/loxP^ mice. Here the number of IRF8^+^ monocytes was significantly lower in the abemaciclib group (Fig. 5B).

In conclusion, prophylactic abemaciclib had a modest long-term effect on bone marrow hematopoiesis in both mouse models. However, the individual effects in each mouse strain provide further evidence for the different response of the two mouse models to abemaciclib.

### *Abemaciclib influences EVs concentration and their procoagulant potential in* Mlh1^−/−^*but not in* Msh2^loxP/loxP^*mice*

**Fig. 6:**
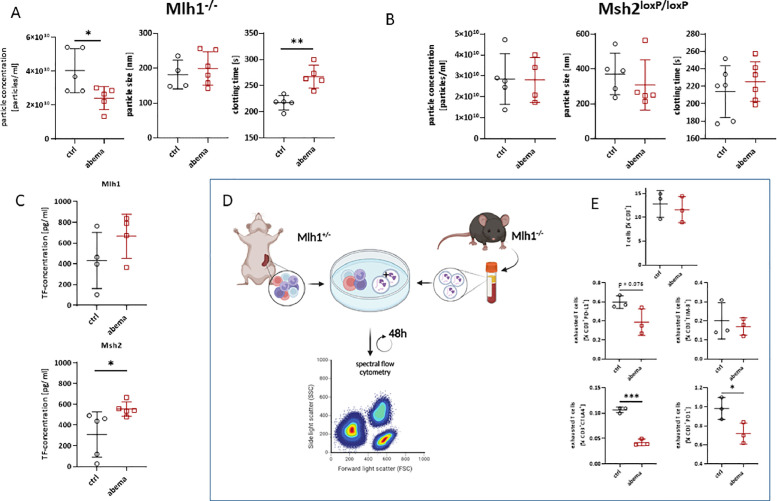
**Characterization, coagulation, and immunosuppressive activity of EVs.** (A,B) EVs were isolated from the plasma of both mouse strains and their concentration and size were analyzed by NTA and the clotting time was determined. (C) Plasma from both mouse strains was analyzed for TF concentration using a TF ELISA kit. (D) Workflow of co-cultivation of EVs and native heterozygous splenocytes of one Mlh1^−/−^ mouse. (E) Detection of the immunosuppressive activity of EVs by spectral flow cytometry. *n* = 3 – 5 mice/group; Mean + SD, * *p* < 0.05, ** *p* < 0.01 *vs*. control; Mann Whitney test.

Plasma EVs from dMMR mice were isolated, quantified and the procoagulant potential evaluated (Fig. 6). Quantification was performed by nanoparticle tracking analysis on NanoSight to analyze the concentration and size of the most abundant EV population. Mlh1^−/−^ mice pretreated with abemaciclib had significantly lower amounts of particles (Fig. 6A). The size of EVs was only marginally affected, but the clotting time was significantly prolonged (*p* < 0.01, Fig. 6A). In Msh2^loxP/loxP^ mice, the concentration of EVs was not affected by abemaciclib. Although there was a slight decrease in size, prolongation of the clotting time was not observed and coagulation times were quite comparable to control mice (Fig. 6B).

Next, we studied the content of TF as primary initiator of extrinsic blood coagulation. Therefore, plasma TF content was determined by ELISA. In both mouse models, abemaciclib increased TF levels, which were even significant in the Msh2^loxP/loxP^ mice (Fig. 6C).

### Abemaciclib prophylaxis reduces the immunosuppressive activity of EVs in Mlh1^−/−^ mice

Finally, the immunomodulatory potential of plasma EVs from control or abemaciclib-treated mice was determined on naïve splenocytes from heterozygous tumor-free, Mlh1 littermates (Fig. 6D, E). The total number of T cells was not significantly different and ranged from 9% to 15%. Hence, we focused on immunomodulatory molecules. Notably, the number of immune checkpoint positive T cells was significantly different between EVs from abemaciclib-treated and control mice (Fig. 6E). In particular, significant changes were observed in CD3^+^ *T* cells expressing CTLA4^+^or PD1^+^. Both checkpoint molecules were downregulated after exposing splenocytes to EVs from abemaciclib-treated mice. In addition, PD-L1 and TIM-3 were lower after exposure to abemaciclib-EVs. However, this did not reach statistical significance.

Hence, EVs from abemaciclib-treated mice alter the phenotype of naïve splenocytes *via* downregulation of immune checkpoint molecules.

In conclusion, we were able to show that dMMR tumors secrete EVs. We could demonstrate that immunomodulatory and eventually tumor-preventing effects of abemaciclib, especially in the Mlh1^−/−^ mouse model, are observable at the subcellular EV level.

## Discussion

This study describes the first prophylactic use of a CDK inhibitor in clinically relevant mouse models of inherited dMMR-related cancers. We showed that repeated administration of abemaciclib significantly delayed tumor development. The later onset of tumorigenesis can be attributed to (I) early immune modulation and preservation of a Th1-driven phenotype, (II) suppression of T-cell exhaustion, (III) reduced ROS production, (IV) altered gene expression, and (V) the secretion of less procoagulant but immunomodulatory EVs. However, strain-specific and individual effects were observed. Mlh1^−/−^ mice benefited more from CDK4/6 inhibition than Msh2^loxP/loxP^ mice. Although this finding is somewhat unexpected, it is consistent with our previous observations in the therapeutic setting [26]. In this earlier study, improved overall survival and effective tumor growth control were seen in Mlh1^−/−^, but not in Msh2^loxP/loxP^ mice. Intrinsic molecular (homozygous *Mlh1* and constitutional *Msh2* knockout) and immunological differences (baseline immune status, degree of immune suppression) may best explain these findings and are consistent with the heterogeneous clinical responses in individual cancer patients [30]. Therefore, patient stratification is an unmet need to improve treatment response and avoid unnecessary side effects.

Despite the differences between Mlh1^−/−^ and Msh2^loxP/loxP^ mice, some overlap after prophylactic CDK inhibition was detectable, especially in the immune response. In particular, the expression profile of immune regulatory molecules such as PD-1 or CTLA-4 and the number of Treg cells were found to be comparable, ultimately delaying tumor development. The ability of abemaciclib in suppressing Treg proliferation while enhancing activation of antigen-experienced T cells was already shown by others [31,32]. Here, we first describe the long-term immunomodulatory effect of abemaciclib in a prophylactic setting. This global immune modulation was evident in both circulating, and splenic T cell subsets, finally contributing to reduced numbers of tumor-infiltrating Tregs and MDSCs.

Hematologic toxicity as a consequence of CDK inhibition is a fact worth mentioning. Previous clinical trials reported low grade neutropenia after abemaciclib administration, compared to both approved CDK4/6 inhibitors palbociclib and ribociclib [1,2,33,34]. This was confirmed in our study as we only observed few alterations in hematopoiesis between individual treatment groups. The only exception was seen for IRF8^+^ monocytes, which were reduced in the bone marrow of Msh2^loxP/loxP^ but not Mlh1^−/-^ mice. Knowing that IRF8 is essential for monocyte differentiation and function, we speculate this finding as one of the potential contributing factors for the final treatment failure in this mouse strain.

Another rationale for abemaciclib in prophylactic and therapeutic settings is the fact that aberrant CDK4/6 function triggers tumor immune evasion and orchestrates an immunologically cold TME [35,36] – regardless of tumor mutational burden. The mouse models used here exemplify this feature, as tumors are barely infiltrated with T cells. This so-called “immune desert” is strongly associated with poor response to immune checkpoint inhibition and represents the clinical counterpart of G2 cancers in Lynch Syndrome [[37], [38], [39]]. Therefore, breaking intrinsic tolerance to immunotherapy is quite challenging. Indeed, we have applied various (combination) strategies in these mice, including vaccination, immune checkpoint inhibition, and CDK blockade, but in most cases, complete tumor regression was not observed [22,23,27,40]. This highlights the need for proper immune activation against these highly aggressive ultra-mutated tumors [41]. Abemaciclib seems quite promising as another study just recently confirmed the ability of this CDK inhibitor in sensitizing otherwise unresponsive tumors to immune checkpoint blocking treatments [42]. In this study, increased antigen presentation and activation of the interferon response pathways was observed in different tumor models.

A deeper analysis on the altered gene expression profile between abemaciclib-treated and control mice revealed a massive increase in neutrophils and mast cells in our study. These latter cells secrete a variety of pro-inflammatory molecules and are involved in intercellular communication with other immune or stromal cells to modulate the immune status or promote tumor progression [43]. Thus, they may play a dual role in cancer regulation. Here, our finding can be interpreted as unfavorable and may provide an explanation for the final tumor progression in both mouse models. In Mlh1^−/−^ mice, this was accompanied by suppression of interferon signaling, and activation of the Hedgehog and Notch signaling pathways. The most deregulated gene was *Gli1*, which is involved in cell proliferation, survival, self-renewal, and invasiveness [44]. In contrast, Msh2^loxP/loxP^ tumors showed activation of the MAPK pathway, specially upregulation of *IL1r2.* IL1R2 is a decoy receptor that neutralizes IL1 and is described to be upregulated in tumor-infiltrating regulatory T cells [45]. Another study even proposed that IL1R2 protects cancer cells from apoptosis. Here, no such correlation was seen, as Msh2^loxP/loxP^ tumors in the abemaciclib group did not have higher numbers of infiltrating Tregs. Activation of the Wnt pathway by abemaciclib, which is commonly observed in the therapeutic setting [26,46,47], was also not seen here. Worth mentioning, however, is the fact that gene expression analysis was done on bulk tumor tissues, without considering heterogeneity in the TME. As this can be either spatial or temporal, more detailed analysis of different tumor regions is warranted.

Additional to local analysis of the TME, circulating biomarkers, such as EVs, are increasingly studied. EVs are secreted from almost all cell types, act as mediators of intracellular communication and are considered early biomarkers of disease [48]. They may even participate in immunomodulatory oncogenic processes, reshape immune function, and are early indicators of cancer-associated thrombosis [[49], [50], [51]]. Here, we studied EVs at the experimental endpoint with a focus on immune modulation and coagulation. Abemaciclib reduced the amount of EV secretion and prolonged the clotting time of EVs in Mlh1^−/−^ mice but not in Msh2^loxP/loxP^ mice, which is in line with the different response to abemaciclib between these two mouse strains. Based on our results, the prolonged clotting time could be explained by the reduced particle concentration. A rather unexpected finding was the higher amount of TF in plasma samples. TF is the physiological activator of the extrinsic coagulation cascade and aberrant TF secretion into the circulation is associated with thrombosis risk in cancer patients [18,52,53]. This risk increases after therapy [18,[54], [55], [56]]. It has been postulated that abemaciclib also increases the risk of thrombosis in breast cancer patients, which may be due to TF release [57]. Hence, our data are partially consistent with these clinical findings, as we also detected higher plasma TF levels in both mouse models. However, this is in contrast to the prolonged clotting time of the EVs. A possible explanation could be that TF is also present in free form in plasma or is expressed by other cell types. However, since direct TF abundance on EVs was not detected, it cannot be excluded that abemaciclib also reduces TF abundance on tumor cells and their secreted EVs. In support of this, no evidence of thrombosis was seen in the mice. Finally, the influence of tumor-derived EVs on the immune system has been demonstrated by several groups [58,59]. However, less is known about whether the immunosuppressive activity of EVs is maintained after therapy, which is why EVs from the endpoint were used. To fully focus on the immunosuppressive activity, the concentrations of EVs were equated, as fewer EVs were secreted in the abemaciclib group. Interestingly, this is contrary to chemotherapeutic agents, which tend to increase EV release, suggesting that the mechanism of EV secretion is already affected by abemaciclib in the cells. Despite different cell types in the blood that are able to secrete EV, no differentiation was made here, as it can be assumed that the exposure in the blood would be comparably low after oral application of abemaciclib. Furthermore, the sole use of tumor-derived EVs would lead to a non-physiological experimental approach, even with low exposure to abemaciclib. The immunomodulatory potential of EVs was investigated in a short-term experimental setting. Specifically, we investigated whether EVs from abemaciclib-pretreated mice have less immunosuppressive activity on naïve splenocytes than EVs from control mice. Here, we focused particularly on T-cell exhausting markers, which can reveal rapid activation of T cells due to their dynamic capabilities. Indeed, we could show that EVs from Mlh1^−/−^ mice reduced the amount of T cell exhaustion markers on naïve splenocytes. One possible explanation is the presence of immune checkpoints on EVs, such as PD-L1. It has already been shown that glioblastoma-derived EVs have PD-L1 on their surface, which inhibits T cell activation via the PD-1/PD-L1 signaling pathway [60]. The excessive presence and co-expression of multiple immune checkpoints lead to increased T cell exhaustion. Here, we could show that EVs from abemaciclib pretreated mice led to a reduction in T cell exhaustion markers, which are therefore, likely less suppressive. Hence, we speculate that pretreatment with abemaciclib exerts long-term immunomodulatory effects characterized by reduced secretion of immunosuppressive EVs from developing tumors as well as altered expressions of cell surface markers that are passed on to the secreted EVs. These, in turn shown an inflamed microenvironment. The low levels of systemic immunosuppression, both at the cellular and subcellular (= EV) levels, and the accompanying reduced procoagulant phenotype renders abemaciclib as a promising preventive agent to delay tumor formation and, eventually, reduce the risk of thromboembolic complications. Our cumulative data from various preclinical in vivo studies [28,40,61,40] confirm the benefit of preventive interventions in selected subgroups of patients who are at high risk of developing tumors. With abemaciclib, we have identified another promising candidate that should be considered for further clinical testing, either alone or in combination with personalized immunomodulatory agents.

## Ethics approval and consent to participate

The German local authority approved all animal experiments: Landesamt für Landwirtschaft, Lebensmittelsicherheit und Fischerei Mecklenburg‐Vorpommern (7221.3‐1‐062/19; −025/20), under the German animal protection law and the EU Guideline 2010/63/EU. All applicable international, national, and/or institutional guidelines for the care and use of animals were followed.

## Consent for publication

“Not applicable”.

## Availability of data and materials

All data generated or analyzed during this study are included in this published article [and its supplementary information files].

## CRediT authorship contribution statement

**Annabell Wolff:** Data curation, Formal analysis, Methodology. **Paula Krone:** Data curation, Methodology. **Johanna Maennicke:** Data curation, Methodology. **Julia Henne:** Data curation, Methodology, Software. **Sonja Oehmcke-Hecht:** Writing – review & editing. **Caterina Redwanz:** Methodology. **Wendy Bergmann-Ewert:** Methodology. **Christian Junghanss:** Writing – original draft. **Larissa Henze:** Writing – review & editing. **Claudia Maletzki:** Conceptualization, Software, Supervision, Validation, Writing – review & editing, Funding acquisition.

## Declaration of competing interest

The authors declare that they have no competing interests.

## Appendix Supplementary materials

Image, application 1
