# Supplementary material for: Prophylaxis with abemaciclib delays tumorigenesis in dMMR mice by altering immune responses and reducing immunosuppressive extracellular vesicle secretion
Source: Transl Oncol. 2024 Jul 9;47:102053. doi: 10.1016/j.tranon.2024.102053 (PMC11296063; doi:10.1016/j.tranon.2024.102053)

**Supplementary Figure 1: Cytokine levels of plasma from Mlh1^-/-^ mice.** Plasma samples were collected before treatment (= day 0), at day 42,and at day 84. Cytokine levels were determined as described in material and methods. Given is the concentration of the indicated marker. n = 4 – 8 mice/group; Mean + SD, * p < 0.05, ** p < 0.01; Mann Whitney test.


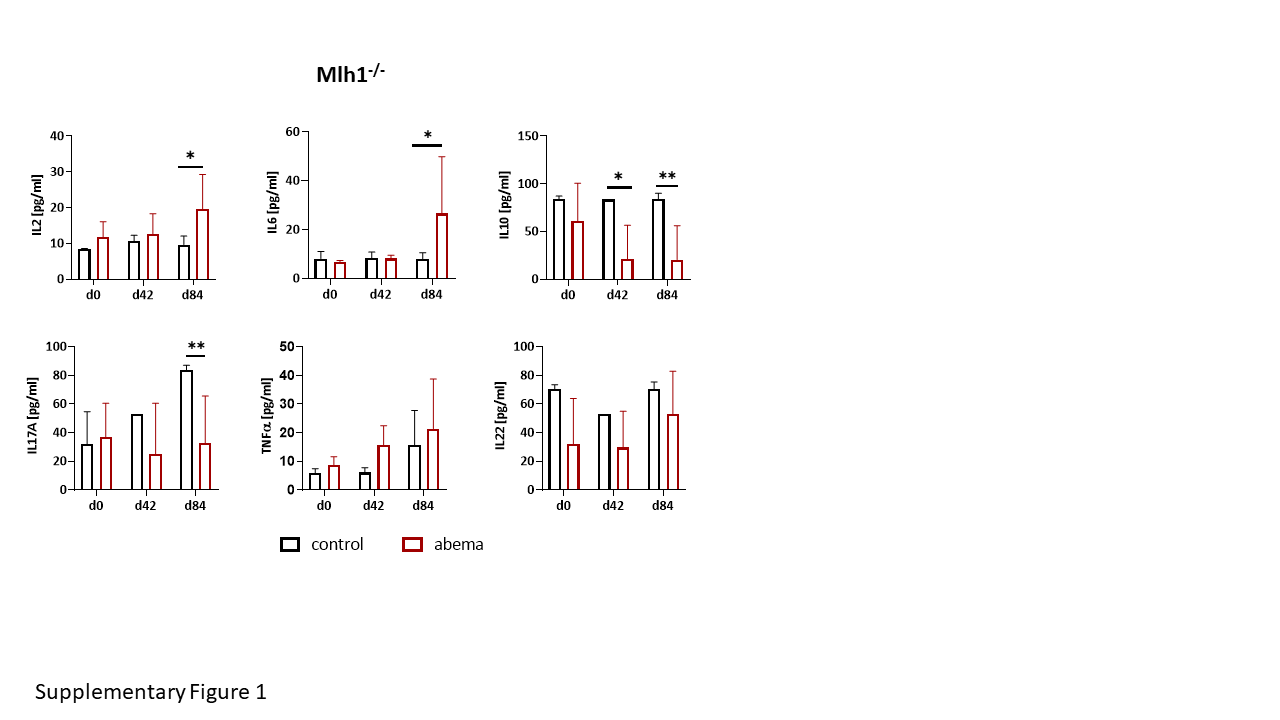


**Supplementary Figure 2: Spectral flow cytometry & Nanostring-based gene expression assay of tumors.** (A) Given is the number of % immune cells at the experimental endpoint resulting from 100,000 events measured on a spectral flow cytometer. Mean + SD. (A) n = 4 – 7 mice/group; (B) The PanCancer IO 360 Gene Expression Panel was applied. Relative abundances measuring various contrasts between cell types reported for each group. Data result from n = 3 samples/group.


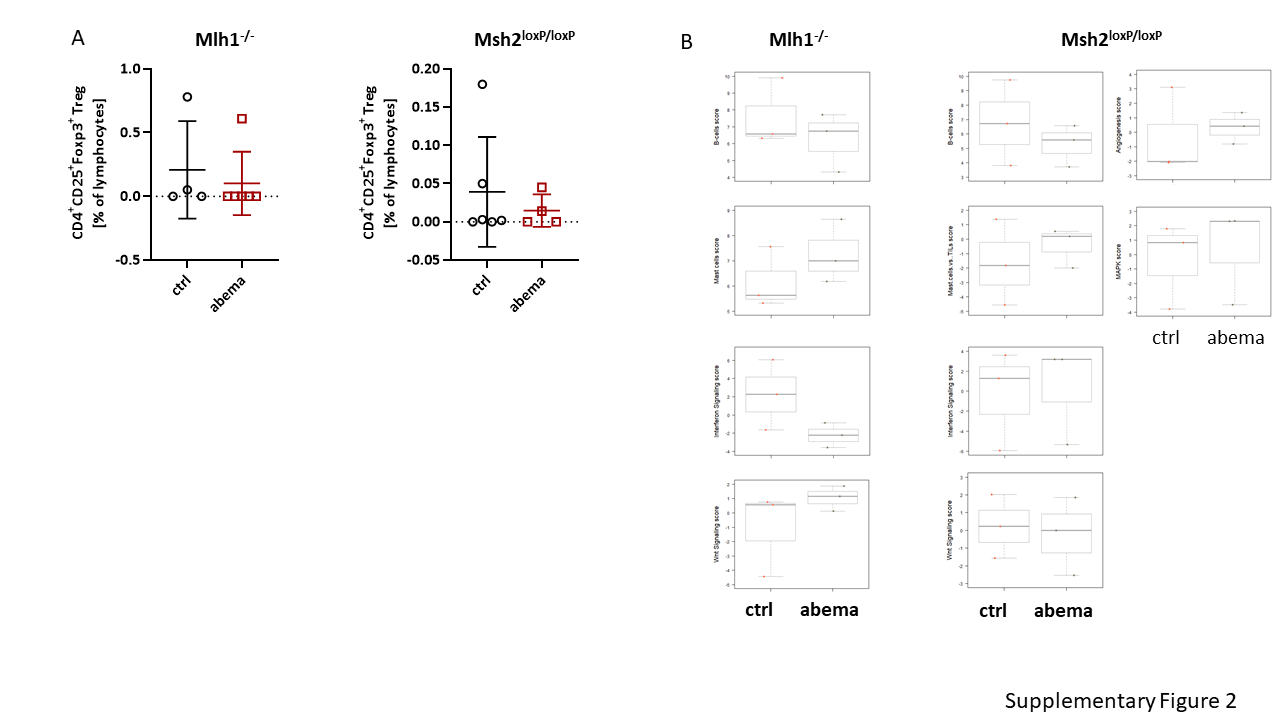

Supplement: Supplementary file 1 [file mmc1.docx]
